# Long-term memory for spatial frequency: a non-replication

**DOI:** 10.1007/s10339-022-01118-w

**Published:** 2022-12-07

**Authors:** Riccardo Sacripante, Sergio Della Sala, Robert H. Logie

**Affiliations:** 1 Human Cognitive Neuroscience, Psychology, University of Edinburgh, Edinburgh, UK; 2 Present Address: Norwich Medical School, University of East Anglia, Norwich, UK

**Keywords:** Long-term memory, Short-term memory, Perceptual memory, Spatial frequency, Psychophysics

## Abstract

Reports on stability of spatial frequency in short-term memory span have confirmed low-level perceptual memory mechanism in early visual processing. However, some studies have also claimed evidence for high-fidelity perceptual long-term storage of spatial frequency. We report an attempted replication of Magnussen et al. (Psychol Sci 14:74–76, 2003) where participants were asked to discriminate the spatial frequency of a reference grating from a test stimulus after intervals of 5 s or 24 h. Group thresholds after 24 h were significantly higher than after 5 s, therefore failing to support long-term storage of spatial frequency.

## Introduction

Several studies have shown that spatial and temporal features of visual images, such as contrast or spatial frequency, are stable in visual short-term memory following 1–30-s retention interval (Regan 1985; Magnussen et al. 1990, 1991; Magnussen and Greenlee, 1992). These findings were deemed to be consistent with a model of short-term memory where dimensions of visual images are coded in parallel representations (Damasio 1989; Magnussen et al. 1990), and, more specifically, with a low-level perceptual memory mechanism in early visual processing (Magnussen and Grenlee 1999; Magnussen 2000).

Whether this storage ability for perceptual properties of visual images is limited to short-term memory storage or could be extended to long-term memory represents a matter of debate. Magnussen and Dyrnes (1994) applied the method of delayed perceptual discrimination (Regan 1985; Magnussen et al. 1990) to assess visual long-term memory with retention intervals up to 50 h. In their study, three reference gratings with different spatial frequencies (2.5, 5.0, 10 cycles per degree—cpd) were followed by a multiple-trial memory test consisting of a series of 11 gratings with a spatial frequency range of ± 1.0 cpd. For every test trial, participants had to judge whether the test grating had a higher or lower spatial frequency compared to the reference grating. This memory test was administered at three fixed time intervals (30 min, 5 h and 50 h). Findings revealed a long-lasting preservation of visual information for sinusoidal gratings up to 50 h, as the functions from the three time intervals overlapped. Magnussen and Dyrnes (1994) interpreted these results in support of high-fidelity spatial long-term memory, as an elementary memory mechanism of pre-categorical storage of visual information or priming (Tulving 1991).

Lages and Treisman (1998) subsequently questioned whether discrimination could be determined by the range of test stimuli used, as explained by the criterion setting theory (Treisman 1984, 1985, 1987). According to this account, the response criterion changes from trial to trial under the influence of those processes prompted to optimize performance (Treisman and Williams 1984; Lages and Treisman 1998). More specifically, the processes involved in discrimination may also include a long-term reference decision criterion. Therefore, each participant’s decision on the delayed discrimination (higher or lower spatial frequency) may have been based on a representation of the entire range of spatial frequencies presented, instead of on a direct comparison to a single frequency (reference stimulus). Indeed, these authors found that 50% of the psychometric functions were determined by the midpoint of the stimulus range. They thus concluded that the findings from Magnussen and Dyrnes’s study (1994) were confounded by the criterion setting of the experiment, meaning that perceptual long-term memory for pre-categorical visual information could be not demonstrated.

As a way of addressing this criticism, Magnussen et al. (2003) carried out a single trial experiment whereby a larger sample of participants (*n* = 166) made a single independent judgement regarding the relative spatial frequency of a single reference grating. By testing participants on this single decision, the effects of criterion setting processes on memory performance could be ruled out. Participants were either tested after a long-term memory interval (24 h) or a short-term memory interval (5 s), considered as control condition. Test trials deviated ± 10% and ± 20% from the reference grating (3 cpd). Their findings confirmed the existence of high-fidelity storage in the long-term memory range, as the psychometric functions for the two time intervals (5 s vs 24 h) did not statistically differ. It was therefore argued that memory representations of abstract visual stimuli can last for days or weeks (Squire and Kandel 1999).

This research design used by Magnussen et al. (2003) was also criticised on the grounds that the discrimination performance may have been influenced by decision processes related to the response criteria, instead of accounting for a visual long-term for spatial frequency (Lages and Paul 2006). It was argued that the illustration of the task (practice trial) on the computer screen may have helped the observers to establish a response criterion before the test trial (Lages and Paul 2006). Moreover, the claim in support of a ‘high-fidelity’ visual long-term memory store rests on affirming a null hypothesis and on the assumption that group performance corresponds to the level of performance for each individual in the group (Ashby et al. 1994; Logie 2018).

Lages and Paul (2006) attempted a partial replication of Magnussen et al. (2003), with the same single trial delayed discrimination task with 5-s and 2-h time intervals to assess the spatial frequency discrimination thresholds. Group performance was also compared with three individual observers who performed the same task but in a repeated trials condition. The practice trial of the task was not presented on the screen but on paper, and hence, participants did not have the chance to establish a response criterion during training. This change in the practice trial may have increased uncertainty about the reference grating. The group discrimination thresholds were significantly higher for 2 h than 5-s retention interval, whereas the thresholds for the 2-h interval obtained from the individual observers appeared considerably lower. Interestingly, the slopes for 5-s and 2-h intervals statistically differed. These findings suggested that discrimination performance depends on task-related information (e.g. response criteria) rather than visual memory of the stimulus (reference grating).

Evidence from psychophysical and neurophysiological research (Reinvang et al. 1998; Magnussen and Greenlee 1999; Magnussen 2000; Magnussen et al. 2009) suggested that elementary attributes of visual stimuli are stored in a subsystem of the Perceptual Representation System (PRS- Tulving and Schacter 1990; Schacter et al. 2000), namely a perceptual mechanism of non-declarative implicit memory (Magnussen 2000). This specific subsystem computes information about global form and structure of visual objects during early visual processing. Further fMRI studies employed the same experimental task to locate the brain regions involved in spatial frequency discrimination of sinusoidal gratings (Greenlee et al. 2000; Sneve et al. 2011).

Given the concerns raised regarding these findings, and the controversy about the existence of a high-fidelity storage in long-term visual memory (Lages and Treisman 1998; Lages and Paul 2006), we here attempted a replication of the original study by Magnussen et al. (2003), based on their experimental design. If the two psychophysical functions for 5-s (control condition) or 24-h (experimental condition) interstimulus interval (ISIs) do not statistically differ, the null hypothesis would be sustained, and the experiment would replicate.

This replication also aimed to address a wider debate: whether or not memory for low-level features such as spatial frequency is effectively retained at longer time intervals of up to 24 h. A replication of the results from Magnussen et al. (2003) would be a promising ground for further investigations including with healthy older adults (see Norman et al. 2014) and patients (see Della Sala et al. 1987; Wilkins et al. 1988).

### Methods

#### Participants

A total of 80 younger adults (55 females and 25 males) between 18 and 47 years of age (*M* = 23.86, SD = 5.36) were recruited from the general population and were tested in the Psychophysics lab of the University of Edinburgh. This initial sample of younger participants was then randomly assigned to two different time interval conditions, either the control condition (5-s ISIs) or the experimental condition (24-h ISIs). Forty participants assigned to the control condition (28 females and 12 males) ranged between 18 and 24 years of age (*M* = 22.02, SD = 3.84). The other 40 participants assigned to the experimental condition (27 females and 13 males) ranged between 18 and 47 years of age (*M* = 25.7, SD = 6.05). As the only requirement to take part in this study, participants needed to have normal or corrected to normal vision. Ethical approval was obtained from the department of psychology research ethics panel of the University of Edinburgh (Ref. No.: 151-1819/5). Participants provided written informed consent.

### Materials

**Fig. 1:**
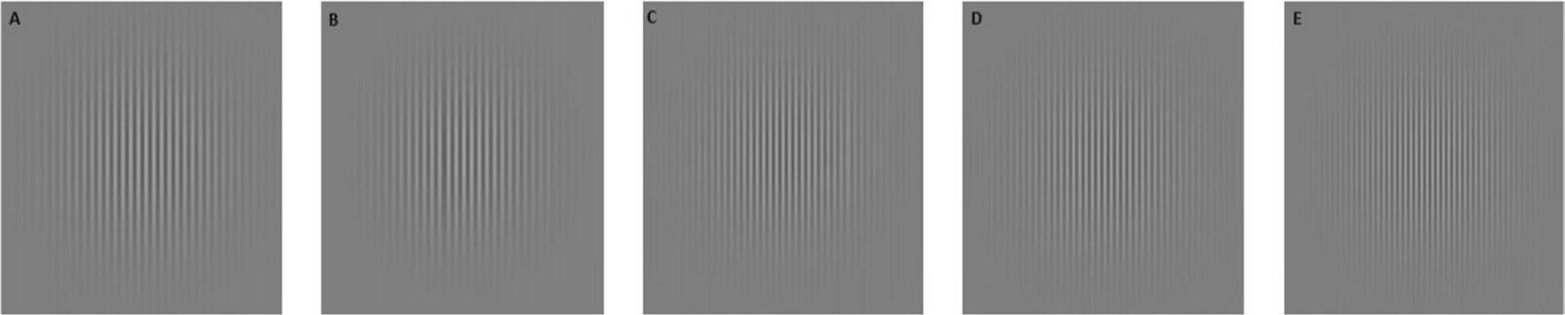
Reference (**C**) and test (**A**, **B**, **D**, **E**) sinusoidal gratings used in the replication study, with approximately 30% contrast. In the figure, the spatial frequency of the test stimuli in (**A**, **E**), respectively, differed from the spatial frequency of the reference stimulus by − 20% and + 20%. The spatial frequency of the test stimuli in (**B**, **D**), respectively, differed from the spatial frequency of the reference stimulus by − 10% and + 10%

As reported by Magnussen et al. (2003), we employed a set of sinusoidal gratings with a contrast of approximately 30% presented on a circular window (Fig. 1) on a Mitsubishi Diamond Pro 930SB monitor screen with a resolution of 2048 by 1536 pixels. The visual angle of the grating field subtended 15°. The spatial frequency of the reference stimulus was set at 3 cycles per degree (cpd), whereas the spatial frequencies of the test trials deviated by ± 10 or ± 20% from the reference stimulus (2.4, 2.7, 3.3, and 3.6 cpd). All the sinusoidal gratings (practice, reference, and test) were presented for a constant time lapse of 5 s.

The distance from the screen was fixed at 57 cm by using a chin rest. The light in the room, placed at 175 cm above the screen, was kept constant too, at the lowest level possible (8.69 Lux with screen on). In addition, the luminosity of the stimuli was measured with a spectroradiometer (Specbos 1201, Jeti) to assess a stable luminosity across all the stimuli; luminosity was not reported by Magnussen et al. (2003), who instead reported the resolution of the screen they used. Stimuli were programmed and presented with PsychoPy 3.

### Procedure

Participants were comfortably seated with their head supported on a chin rest located at 57 cm away from the computer screen. The experimental procedure was explained to participants and the task was illustrated by showing a practice trial consisting of two gratings. One grating had 2 cpd (lower spatial frequency) and the other had 5 cpd (higher spatial frequency), and each was displayed for 5 s, with an ISI showing a fixation point for 5 s. They were then asked to judge whether the second grating had higher or lower spatial frequency than the first grating by pressing Z or M on the keyboard. The allocation of the keys to ‘higher’ (thinner lines) or ‘lower’ (thicker lines) was counterbalanced. This served as a practice trial and the difference between the two gratings was sufficiently large to make the difference obvious and hence the task understandable.

After a brief pause, they were presented with the reference grating (3 cpd) for 5 s which they were to examine carefully. After a 5-s or 24-h ISI (fixation point on the screen), a single test grating was presented. The test grating differed ± 10% (3.3 or 2.7 cpd) or ± 20% (3.6 or 2.4 cpd) from the reference frequency. Participants had to decide whether this second grating had higher or lower spatial frequency than the reference grating by pressing Z or M on the keyboard. The allocation of the keys to ‘higher’ (thinner) or ‘lower’ (thicker) was counterbalanced.

### Results

A 2 × 4 between-subjects design was used, with two independent groups of participants assigned to a time interval (ISI-5 s or 24 h) and four relative spatial frequency conditions (± 10 or ± 20 from the reference stimulus). The two groups of participants (40 in each group) were exposed to the same practice trial prior to the experiment and to the same reference and test grating (depending on the relative spatial frequency they were assigned to). As in the original Magnussen et al. (2003) study, the outcome measure was the proportion of ‘higher’ judgements. More specifically, ‘higher’ and ‘lower’ judgements, respectively, corresponded to ‘thinner’ and ‘thicker’ bars.

All the participants were tested individually, with study and test conditions separated by an interval of 5 s (control condition) for half (40) of the participants or 24 h for the remaining participants (time of the day was the same for study and test for each participant, but varied across participants, according to their availability).

Following the analysis procedure reported by Magnussen et al. (2003), it was attempted to fit each data set into Weibull psychometric functions using a constrained maximum likelihood fit (Wichmann and Hill 2001) with the quickpsy package (Linares et al. 2016) in R (version 4.0.3). The response and the corresponding proportion of higher judgements are reported in Table 1.

**Table 1:** Judgements made by participants in the 5-s (above) and 24-h (below) ISI intervals, according to the relative spatial frequency condition they were assigned to

Relative spatial frequency	ISI	N thinner	N thicker	Proportion of higher judgements
0.8	5 s	1	9	0.1
0.9	5 s	5	5	0.5
1.1	5 s	5	5	0.5
1.2	5 s	9	1	0.9
0.8	24 h	6	4	0.6
0.9	24 h	8	2	0.8
1.1	24 h	4	6	0.4
1.2	24 h	6	4	0.6

**Fig. 2:**
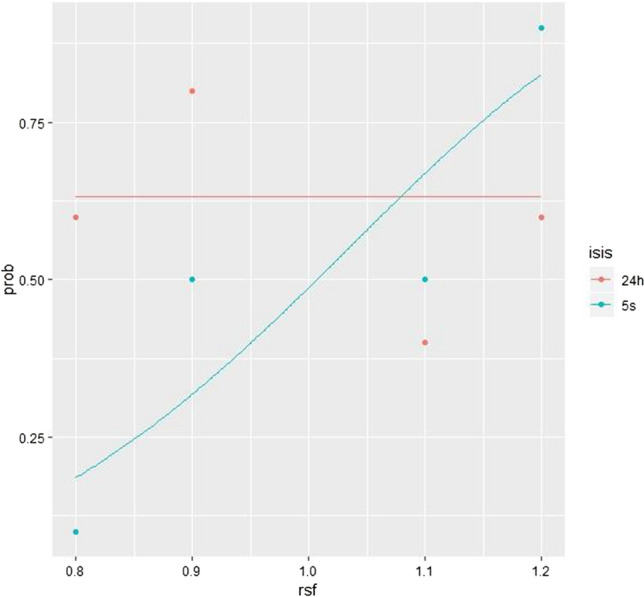
This figure illustrates the proportion (prob) of participants who guessed that the test trial has a higher spatial frequency (‘thinner bars’) compared to the reference grating. These findings are illustrated as a function of the relative spatial frequency (rsf; Weber fractions, test frequency/reference frequency) of the test grating and the ISIs (24 h vs. 5 s). Each point represents 10 participants. These data were fitted into Weibull functions

Data from the 5 s condition successfully fit into a Weibull function, while data from 24-h ISIs condition failed to do so (see Fig. 2). Weibull function fits were indicated by the group thresholds calculated from the functions, which ranged from 0.92 to 1.10 for 5 s and from 0.0 to 0.95 for 24 h, with an overall difference of –1.00 (ranging from − 1.10 to − 0.64). Therefore, group thresholds were significantly and disproportionately larger for 24 h than for 5 s, which resulted in a poorer Weibull function fit for these data.

To evaluate whether the distributions were statistically different, Magnussen et al. (2003) performed chi-squared tests based on delta values obtained pooling correct and incorrect responses for trials with ± 10 deviation and trials with ± 20 deviation, respectively. We used the same statistical approach so our replication could adhere to the criteria specified by Magnussen et al. (2003). Thus, for the 20 observations made in each test manipulation, the number of correct and incorrect responses was calculated for each ISIs. For the 10% manipulation, the chi-squared test revealed a value of 1.6, *p* = 0.2, while, for the 20% manipulation, the chi-squared revealed a value of 6.4, *p* = 0.011. In contrast with Magnussen et al. (2003), our results suggest a significant difference between the two psychometric functions for the ± 20% experimental condition.

## Discussion

In the present experiment, we attempted a systematic replication of the experimental procedure devised by Magnussen et al. (2003) in support of the existence of a ‘high-fidelity’ perceptual long-term memory. As noted in the introduction the evidence for such a memory ability is inconsistent. Both the experimental design and the response criteria on the delayed discrimination task may have been confounds in generating the participants’ performance in those previous studies.

In our attempted replication, the group discrimination thresholds significantly differed between the two ISIs groups (5 s vs 24 h), as the spatial frequency discrimination thresholds were markedly larger after the 24 h test separation. In addition, the null hypothesis was rejected for one of the experimental manipulations (± 20%), which is enough to conclude that the proportion of higher judgements significantly differed between the two ISIs. As such, these findings do not seem to support the evidence for a ‘high-fidelity’ perceptual long-term memory.

Overall, the proportion of higher judgements in the 24 h condition did not fit into a Weibull function, meaning that these data did not seem to follow the pattern previously observed by Magnussen et al. (2003). Indeed, more than half of the participants assigned to the 24 h condition (24/40) failed to correctly discriminate the test trial from the reference grating in both the experimental manipulations. Instead, participants assigned to the 5-s ISI condition correctly discriminated the test trial from the reference only in the ± 20% manipulation, while those assigned to the ± 10% experimental manipulation condition appeared to respond by chance (0.5/0.5).

Together with other previous reports (see Lages and Paul 2006), these findings cast doubt on the existence of a high-fidelity perceptual long-term memory. Although the allocation of the keys (Z or M) to ‘higher’ (thinner) or ‘lower’ (thicker) was counterbalanced, a response criterion could have been driven by chance.

Nonetheless, the sample included in this report may have influenced the outcome of the replication, as it amounted to less than half of the number of participants (*n* = 166) originally considered by Magnussen et al. (2003). Indeed, the loss in power reflected in poorer fit to the Weibull function, as seen with data in 24-h ISI group, where the psychometric function was based on 10 responses rather than 20 as in Magnussen et al. (2003). Crucially, the smaller sample size limited the power of the comparison between the 24-h and 5-s ISI conditions. In the 5-s ISI group the level of noise was still considerably high, especially in the participants tested with ± 10% experimental manipulation condition.

Nevertheless, in the original study, the effect size was not indicated, making it harder to estimate a sufficient number of participants to be tested. So, the effect size may be very small, and so require large numbers of participants to be detected. Furthermore, the age range of our sample may have been broader than the original sample, although Magnussen et al. (2003) did not report the age range of the participants included in their study but only their background (undergraduate students).

Although in the field of psychophysics and cognitive psychology there is compelling evidence in support of short-term memory for spatial frequency, the issue of whether there is a long-term aspect of perceptual memory for spatial frequencies was not resolved nor addressed in previous studies. Indeed, the experimental paradigm devised by Magnussen et al. (2003) is not free from confounding factors that may have potentially influenced the results (see Lages and Paul 2006).

Future studies may consider the use of more complex perceptual stimuli to test for high-fidelity visual long-term memory in the absence of a meaningful context. However, consistent with Lages and Paul (2006), our results suggest that the evidence for a high-fidelity long-term perceptual memory for low-level, non-meaningful visual features such as spatial frequency might not be robust.
